# Buruli Ulcer, Nigeria

**DOI:** 10.3201/eid1305.070065

**Published:** 2007-05

**Authors:** Okechukwu Chukwuekezie, Edwin Ampadu, Ghislain Sopoh, Ange Dossou, Alexandre Tiendrebeogo, Lola Sadiq, Françoise Portaels, Kingsley Asiedu

**Affiliations:** * Federal Ministry of Health, Abuja, Nigeria; † Ministry of Health, Accra, Ghana; ‡ Centre de Dépistage et de Traitement de l'ulcère de Buruli d’Allada, Cotonou, Benin; § World Health Organization, Libreville, Gabon; ¶ World Health Organization, Abuja, Nigeria; # Institute of Tropical Medicine, Antwerp, Belgium; ** World Health Organization, Geneva, Switzerland

**To the Editor:** Buruli ulcer (BU), a neglected tropical disease caused by *Mycobacterium ulcerans*, is characterized by necrosis of subcutaneous tissue, leading to chronic, painless, and progressive ulcers. Without proper treatment, BU results in severe and permanent disability in more than a quarter of patients. Most patients are children <15 years of age. BU has been reported in >30 countries (*1*). The World Health Organization (WHO) has described the epidemiology, clinical features, diagnosis, and treatment of BU (*1**–**3*).

In 1967, Gray et al. described 4 BU cases in the Benue River Valley in Nigeria (*4*). The authors also reported unpublished cases of the disease in Banbur, Adamawa State, in the upper part of the Benue River Valley. In 1976, Oluwasanmi et al. described 24 BU cases in and around Ibadan (*5*). Since then, there has been no official report of BU in Nigeria. However, unofficial reports indicate that the disease is still present in the country. For example, between 1998 and 2000, BU cases from the Leprosy and Tuberculosis Hospital in Moniaya-Ogoja, Cross River State, were bacteriologically confirmed at the Institute of Tropical Medicine in Belgium (*6*). More recently, patients from Nigeria have been treated in the neighboring countries of Benin (*7*) and Cameroon (*8*).

To clarify the BU situation in Nigeria, the government, with technical assistance from WHO, carried out a rapid assessment in the southern and southeastern states of the country, where cases had been previously reported. Preassessment sensitization workshops for health workers within the selected states were held in June and July 2006. The assessment took place November 15–19, 2006. The team, which was made up of international experts and national and state health officials, was divided into 2 groups. Group A visited Akwa Ibom and Cross Rivers States, and group B visited Anambra, Ebonyi, and Enugu States.

Based on the WHO case definitions (*1*), 14 of 37 patient examined were considered likely to have BU (9 active and 5 inactive cases); 9 were children <15 years of age. Eight patients were female, and 6 were male. One of the patients with active disease had the edematous form, 1 had osteomyelitis and ulcer, and the other 7 had ulcers (Figure). Ten of the patients had lesions on the lower limbs, 3 on the upper limbs, and 1 on the face. All cases were documented by registration on a modified version of the BU 02 form (*1*) and photography. Swab specimens were taken from all active ulcerative lesions. A fine-needle aspiration technique was used to obtain specimens from the edematous patient. In 4 (44%) of the 9 patients with active cases, the clinical diagnosis was confirmed by the IS2404 PCR at the Institute of Tropical Medicine.

**Figure:**
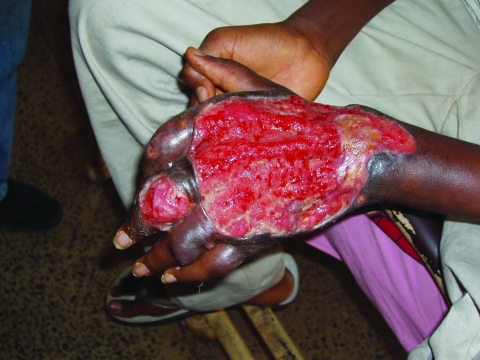
A typical Buruli ulcer in a 17-year-old boy identified during the assessment.

The locations and number of cases identified in each are as follows: Ifite Ogwari village, Ayamelum Local Government Area (LGA), Anambra State (4 cases); Ndo Etok village, Ogoja LGA, Cross River State (3 cases); Nkpo Hamida village, Igbo-Eze North LGA, Enugu State (1 case); Iburu village, Ohaozora LGA, Ebonyi State (1 case); Akofu village, Ikwo LGA, Ebonyi State (1 case); Amagunze village, Nkanu East LGA, Enugu State (1 case); Okro Mbokho village, Eastern Obolo, Akwa Ibom State (1 case); Oron village, Oron LGA, Akwai Ibom State (1 case); and Ugwu Tank, Awka South LGA, Anambra State (1 case).

In conclusion, 30 years after the last publication (*5*) of cases in southwestern Nigeria, BU cases have been found in the southern and southeastern and parts of the country. A similar phenomenon occurred in Cameroon, where a case search in 2001 in 2 districts where cases had last been reported 24 years earlier found 436 active and inactive cases (*8*). These findings demonstrate that BU has not disappeared from Nigeria and that the absence of any regular reporting should be investigated. Although the assessment team was able to visit only 5 of the 36 states (8 of 774 LGAs and 10 communities), it concluded that BU is still present in Nigeria and may be more prevalent than had been previously thought. The lack of familiarity with the disease by health workers may also have contributed to poor reporting.

The assessment team recommended 5 measures: 1) inclusion of BU treatment and control activities in the Tuberculosis and Leprosy Control Program at federal, state, and LGA levels to enhance surveillance of the disease; 2) training of health workers at all levels; 3) a detailed assessment of the extent of BU in the 5 states visited as well as in other states; 4) approaching partners supporting tuberculosis and leprosy control activities in Nigeria to include BU; and 5) incorporating BU into the national surveillance system to allow better data collection.
